# Revealing a word superiority effect using a unique variant of the Latin alphabet: the evidence from Turkish

**DOI:** 10.3389/fpsyg.2024.1367891

**Published:** 2024-05-23

**Authors:** Timothy R. Jordan, Aleynanur Kalan

**Affiliations:** Department of Psychology, Ibn Haldun University, Istanbul, Türkiye

**Keywords:** word perception, word recognition, Turkish, Reicher-Wheeler paradigm, visual perception

## Abstract

When visual stimuli are presented briefly, words are perceived better than nonwords. It is widely accepted that this word superiority effect reflects the efficiency with which words are perceived. However, most of what is known about the effect comes from languages (like English) using the basic Latin alphabet and little is known about whether languages using an alphabetic variant with very different properties can also produce word superiority. Here we report an experiment in which words and nonwords were presented briefly in Turkish, which uses a unique variant of the Latin alphabet containing 29 separate letters, 12 of which are close visual replications of other letters. Despite the potential for visual confusability and perceptual uncertainty, the findings revealed a clear advantage for words over nonwords, indicating that word superiority observed previously for the Latin alphabet can also be observed with the very different variant of this alphabet used for Turkish. Implications of these findings for processes involved in visual word perception are discussed.

## Introduction

For many years (e.g., Cattell, 1886; Pillsbury, 1897), empirical investigations of the perceptibility of alphabetic stimuli have inspired considerable interest in how the physical characteristics of words are processed. One particularly influential finding, reported by Cattell and subsequently replicated in numerous studies (e.g., Reicher, 1969; Wheeler, 1970; Baron and Thurston, 1973; Jordan et al., 2000), is that, when stimuli are presented very briefly, people perceive real words better than nonwords. This phenomenon, called “the word superiority effect,” suggests that, when viewing time is limited, the physical characteristics of words produce a more perceptible stimulus than nonword letter strings. Moreover, crucial research using the two-alternative Reicher-Wheeler task (after Reicher, 1969; Wheeler, 1970) to suppress influences of guesswork which may artifactually advantage words (e.g., Reicher, 1969; Wheeler, 1970; see also Johnston, 1978), indicates this difference in performance between words and nonwords can be attributed to differences in the *perception* of each stimulus type (word, nonword) rather than to differences in the effectiveness of non-perceptual guesswork.

Most of what is known about the word superiority effect comes from research conducted in languages (mostly English) using a basic form of the Latin alphabet, but little is known about whether languages using a variant of the Latin alphabet with very different visual properties also produce a word superiority effect. Turkish is the most widely spoken of the Turkic languages, with around 100 million speakers worldwide. But the Turkish language is relatively modern, derived from the Latin alphabet and introduced in 1928 as part of Atatürk’s reforms in the early years of the Republic of Türkiye. This new alphabet replaced the Ottoman Turkish alphabet (a form of the Perso-Arabic script) with a version of the Latin alphabet which uses 17 distinct letters and a further 12 letters derived from 2 versions of each of 6 base letters: lower case c ç g ğ ı i o ö s ş u ü; upper case C Ç G Ğ I İ O Ö S Ş U Ü. Thus, the Turkish alphabet comprises 29 separate letters, 12 of which are very close visual replications of other letters in the alphabet.

This widespread visual similarity between letters may cause general processing difficulty when recognizing words due to perceptual uncertainty produced by inter-letter confusability. Indeed, the same base letters with and without diacritics are given full and separate letter status in Turkish; for example, the word köşe (meaning *corner*) would form a very different word if perceived as köse (meaning *without facial hair*), and the word ağır (meaning *heavy*) would form a nonword if perceived as agır. But the precise effects of diacritical variations on word recognition in Turkish are currently unclear and such effects may indeed vary across different languages. For example, using a letter priming task, Chetail and Boursain (2019) concluded that, in French, diacritic letters (e.g., à) are processed as separate letter identities from their base letter (a). However, Perea et al. (2019) conducted a similar study in Spanish and concluded that diacritic letters (e.g., é) and their base letter (e) are processed as the same letter identities and that both forms can be considered as variants of the same letter representations (although Perea et al. also found some evidence that diacritic and base letters are processed as separate letter identities). More recently, Benyhe et al. (2023) used a word priming task for Hungarian words and found that word primes with an omitted diacritic were just as effective as same identity primes whereas the addition of a diacritic to a word prime slowed down the processing of word targets (see also Kinoshita et al., 2021).

But other evidence specifically for Turkish and obtained using a different phenomenon (Repetition Blindness, where repetition of identical letters across two brief displays impairs their perception), suggests that Turkish readers classify diacritic and non-diacritic versions of a Turkish letter as variants of the *same* letter, even though each variant of a letter will ultimately distinguish between separate lexical items (Ayçiçeği and Harris, 2002). Thus, words like köşe and köse may initially be processed as having the same 4 letter types, but different lexical entries will eventually become activated as letter perceptions are refined, and different words will eventually be perceived.

Against this rather complex background, it is not yet clear how the high level of letter confusability that exists across the Turkish alphabet may affect the actual perception of Turkish words and, moreover, how Turkish word perception generally may function under the brief presentation conditions required for a word superiority effect to occur. In particular (and following the findings and arguments of Ayçiçeği and Harris, 2002), the considerable letter similarity that exists across the Turkish alphabet raises the possibility that perception of Turkish words generally is less efficient than in languages using the Latin alphabet (e.g., English) due to general perceptual uncertainty produced by multiple letter similarities. Indeed, this effect of inter-letter confusability in the Turkish alphabet may be more damaging for perception of briefly presented words than nonwords because slow or inaccurate processing of letters may greatly disrupt access to lexical entries for words whereas nonwords have no such lexical entries to which access can be similarly impaired. But little is known about the processes underlying the efficiency of Turkish word perception and, in particular, it remains to be seen whether Turkish words are even capable of producing a word superiority effect. Accordingly, the purpose of this study was to establish whether a word superiority effect could be obtained using the Turkish alphabet.

## Methods

### Participants

Thirty-four adults (mean age 22 years) participated in the experiment. All were native speakers of Turkish and had normal or corrected-to-normal vision as determined by a Snellen eye chart. All participants were informed about the experimental procedures and gave written consent before the experiment, according to the Declaration of Helsinki.

### Stimuli

Ninety-six pairs of four-letter Turkish words (with a mean frequency of written occurrence of 105 per million; Ölker, 2011), were selected as experimental stimuli. In line with the requirements of the Reicher-Wheeler task, members of each pair differed by just one “critical” letter (e.g., esin, esir), and critical letters appeared equally often in each of the four possible letter positions. Ninety-six pairs of nonwords were formed by rearranging the non-critical letters in each word pair to form a matched pair of nonword letter strings which do not exist as words in Turkish (e.g., iesn, iesr) but which shared the same critical letters in the same letter positions as the word pairs from which they were formed. All stimuli were presented in lower case Arial typeface. An additional 40 word pairs and 40 nonword pairs were constructed to provide 80 practice stimuli at the beginning of the experiment. For each trial, a different pattern mask was constructed from pseudo randomly arranged fragments of the letters used in the character set, with the constraint that no letters were formed from these fragments. Four proportionally spaced letter *x*s subtended a visual angle of approximately 1.10° horizontally and 0.25° vertically and each mask subtended a visual angle of approximately 1.25° horizontally and 0.40° vertically.

### Apparatus

The experiment was controlled using a Dell 11th Gen Intel(R) Core i7-11700 2.50GHz processor running PsychoPy (v3.8) experiment control software. Stimuli were presented on a UHD 27-inch monitor at a resolution of 2,560 × 1,440 and a refresh rate of 240 Hz. Participants entered their responses via two keys on a MilliKey Response Box interfaced with the computer.

### Design and procedure

Participants took part in a single session. All experimental stimuli were presented randomly to all participants. At the start of each trial, a small fixation point appeared at the center of the screen. When participants pressed a key, the fixation point disappeared, and the following display sequence was initiated: 500 ms blank; stimulus; mask; 500 ms blank. Four dashes were then presented, corresponding to the four letter positions in a four-letter stimulus. At one of these dashes, two letters were shown, one above the dash and one below, and participants had to decide which of these two letters had been present in the stimulus at the position indicated by the dash. To make their choice, participants pressed one of two response keys to select either the upper or lower alternative. Throughout the practice and experimental sections, each sequence (cycle) of 16 trials contained equal numbers of words and nonwords across the four critical letter positions, selected pseudorandomly. Exposure durations were reassessed for each participant after each cycle. Within a cycle, all stimuli were shown for the same exposure duration and when adjustments to exposure duration were made, the same adjustment was made for all stimulus conditions. This procedure ensured that overall performance fell within the midrange of the performance scale for each participant and that each condition (words, nonwords, critical letter positions) was represented equally at the same exposure durations (for further details of these procedures, see Jordan and Bevan, 1996; Jordan et al., 2000). Mean exposure durations for experimental stimuli ranged from 25.00 to 45.83 ms across participants; masks were always presented for 100 msec longer than each stimulus.

## Results

**Figure 1:**
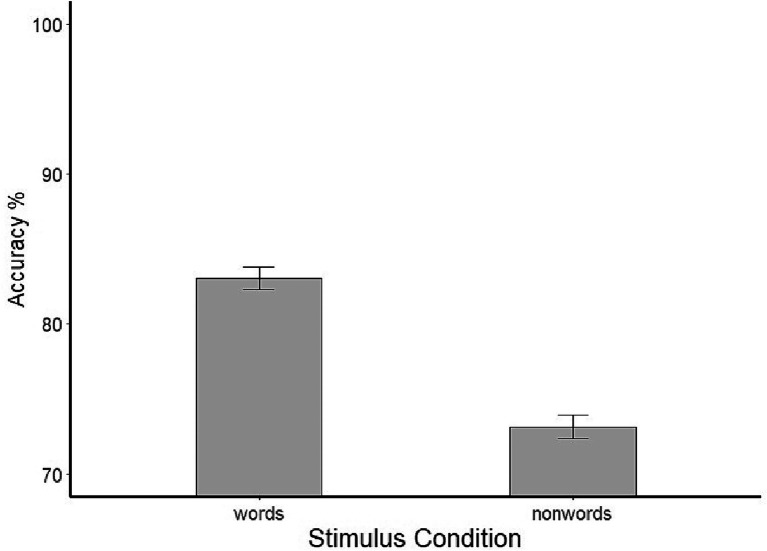
Mean percentage of critical letters correctly reported for words and nonwords. Error bars represent standard errors.

The means for each stimulus condition are shown in Figure 1. A paired samples *t*-test showed a highly significant difference between accuracy rates for words (*M* = 83.01; *SD* = 3.73) and nonwords (*M* = 73.14; *SD* = 4.03), *t*(33) = 9.641, *p* < 0.0001, indicating that performance was more accurate for words.

## Discussion

The purpose of this experiment was to establish whether a word superiority effect could be obtained using the Turkish alphabet where inter-letter confusability and perceptual uncertainty may damage the general efficiency of Turkish word perception. The findings revealed a clear advantage for words over nonwords, indicating that a word superiority effect observed previously for languages using the Latin alphabet can also be observed for the very different variant of the Latin alphabet used for Turkish. Research into reading Turkish is developing (e.g., Özkan et al., 2021; Acartürk et al., 2023), and these new findings provide an important new basis for revealing the processes underlying Turkish word perception. In particular, as word superiority effects are likely to reflect the relative efficiency with which visually presented words are processed, the findings indicate that this efficiency for word perception occurs despite the widespread visual letter similarity that exists across much of the Turkish alphabet, and the disruptive effects this may have generally on perception of Turkish words. Indeed, this evidence of word superiority for Turkish was obtained under the very brief viewing conditions typically used to reveal a word superiority effect, where the temporal efficiency with which words are perceived is likely to be of considerable importance.

The precise nature of Turkish word perception remains to be determined but the finding of a word superiority effect in this language offers new insights. For example, and in line with our previous comments, despite visual letter similarities across the Turkish alphabet, the processing of words appears to be sufficiently rapid to support word perception even when displays of stimuli are extremely brief and post masked. Thus, notwithstanding the notion that Turkish word perception may involve a relatively complex process of letter analysis due to general perceptual uncertainty (see Ayçiçeği and Harris, 2002), the activation of processes relevant to word perception appears, nevertheless, to occur sufficiently rapidly to produce a word superiority effect. But the inter-letter visual similarity present in the Turkish alphabet may also inspire a general use of features in Turkish word perception that are greater than individual letters, derived, for example, from supra-letter or configural analyses of letter groups and whole words (e.g., Patching and Jordan, 2005; Allen et al., 2009; Jordan et al., 2016a,b). For example, the words köşe and köse differ not only in their letter content but also in their overall shapes, and this relatively coarse information may provide important visual cues to word identity. Indeed, diacritics in Turkish are consistently placed on the outer extremities of letters, where their visibility may influence greatly the overall shapes of letter groups and whole words. Further research will help reveal the influences of these features on Turkish word perception generally and on the word superiority effect itself. But the indication now is that a word superiority effect can indeed be obtained even with the unique variant of the Latin alphabet used for Turkish which differs substantially from the ubiquitous form of the Latin alphabet used in many other languages.

## Data availability statement

The raw data supporting the conclusions of this article will be made available by the authors, without undue reservation.

## Ethics statement

The studies involving humans were approved by Ibn Haldun University Ethics Committee. The studies were conducted in accordance with the local legislation and institutional requirements. The participants provided their written informed consent to participate in this study.

## Author contributions

TJ: Conceptualization, Funding acquisition, Investigation, Methodology, Project administration, Supervision, Writing – original draft, Writing – review & editing, Data curation, Formal analysis, Resources, Software, Validation, Visualization. AK: Formal analysis, Methodology, Writing – original draft, Writing – review & editing, Software, Resources, Investigation, Conceptualization, Data curation, Project administration, Validation.

## References

[ref1] Acartürk C. Özkan A. Pekçetin T. N. Ormanoğlu Z. Kırkıcı B. (2023). TURead: an eye movement dataset of Turkish reading. Behav. Res. Methods 56, 1793–1816. doi: 10.3758/s13428-023-02120-6 37450220 PMC10991032

[ref2] Allen P. A. Smith A. F. Lien M. C. Kaut K. P. Canfield A. (2009). A multistream model of visual word recognition. Atten. Percept. Psychophys. 71, 281–296. doi: 10.3758/APP.71.2.281, PMID: 19304618

[ref3] Ayçiçeği A. Harris C. L. (2002). How are letters containing diacritics represented? Repetition blindness for Turkish words. Eur. J. Cogn. Psychol. 14, 371–382. doi: 10.1080/09541440143000113

[ref4] Baron J. Thurston I. (1973). An analysis of the word superiority effect. Cogn. Psychol. 4, 207–228. doi: 10.1016/0010-0285(73)90012-1, PMID: 38544152

[ref5] Benyhe A. Labusch M. Perea M. (2023). Just a mark: diacritic function does not play a role in the early stages of visual word recognition. Psychon. Bull. Rev. 30, 1530–1538. doi: 10.3758/s13423-022-02244-4 36635587

[ref6] Cattell J. M. (1886). The time taken up by cerebral operations. Mind XI, 220–242. doi: 10.1093/mind/os-XI.42.220

[ref7] Chetail F. Boursain E. (2019). Shared or separated representations for letters with diacritics? Psychon. Bull. Rev. 26, 347–352. doi: 10.3758/s13423-018-1503-0, PMID: 29987764

[ref8] Johnston J. C. (1978). A test of the sophisticated guessing theory of word perception. Cogn. Psychol. 10, 123–153. doi: 10.1016/0010-0285(78)90011-7, PMID: 668297

[ref9] Jordan T. R. Bevan K. M. (1996). Position-specific masking and the word-letter phenomenon: Reexamining the evidence from the Reicher-Wheeler paradigm. J. Exp. Psychol. Hum. Percept. Perform. 22, 1416–1433.

[ref10] Jordan T. R. Dixon J. McGowan V. A. Kurtev S. Paterson K. B. (2016a). Fast and slow readers and the effectiveness of the spatial frequency content of text: Evidence from reading times and eye movements. J. Exp. Psychol. Hum. Percept. Perform. 42, 1066–1071. doi: 10.1037/xhp0000234, PMID: 27123680

[ref11] Jordan T. R. Dixon J. McGowan V. A. Kurtev S. Paterson K. B. (2016b). Effects of spatial frequencies on word identification by fast and slow readers: Evidence from eye movements. Front. Psychol. 7:1433. doi: 10.3389/fpsyg.2016.01433, PMID: 27733837 PMC5039934

[ref12] Jordan T. R. Patching G. R. Milner A. D. (2000). Lateralized word recognition: Assessing the role of hemispheric specialization, modes of lexical access and perceptual asymmetry. J. Exp. Psychol. Hum. Percept. Perform. 26, 1192–1208. doi: 10.1037//0096-1523.26.3.1192, PMID: 10884017

[ref13] Kinoshita S. Yu L. Verdonschot R. G. Norris D. (2021). Letter identity and visual similarity in the processing of diacritic letters. Mem. Cognit. 49, 815–825. doi: 10.3758/s13421-020-01125-2, PMID: 33469882 PMC7614445

[ref14] Ölker G. (2011). Yazılı Türkçenin kelime sıklığı sözlüğü (1945–1950 arası) (Doctoral dissertation, Selçuk University).

[ref15] Özkan A. Beken Fikri F. Kırkıcı B. Kliegl R. Acartürk C. (2021). Eye movement control in Turkish sentence reading. Q. J. Exp. Psychol. 74, 377–397. doi: 10.1177/1747021820963310, PMID: 32976053

[ref16] Patching G. R. Jordan T. R. (2005). Spatial frequency sensitivity differences between adults of good and poor reading ability. Invest. Ophthalmol. Vis. Sci. 46, 2219–2224. doi: 10.1167/iovs.03-1247, PMID: 15914644

[ref17] Perea M. Fernández-López M. Marcet A. (2019). What is the letter é? Sci. Stud. Read. 24, 434–443. doi: 10.1080/10888438.2019.1689570

[ref18] Pillsbury W. B. (1897). A study in apperception. Am. J. Psychol. 8, 315–393. doi: 10.2307/1411485, PMID: 38176480

[ref19] Reicher G. M. (1969). Perceptual recognition as a function of meaningfulness of stimulus material. J. Exp. Psychol. 81, 275–280. doi: 10.1037/h0027768, PMID: 5811803

[ref20] Wheeler D. D. (1970). Processes in word recognition. Cogn. Psychol. 1, 59–85. doi: 10.1016/0010-0285(70)90005-8, PMID: 38616615

